# Jasmonate signalling drives time‐of‐day differences in susceptibility of Arabidopsis to the fungal pathogen *Botrytis cinerea*

**DOI:** 10.1111/tpj.13050

**Published:** 2015-11-21

**Authors:** Robert A. Ingle, Claire Stoker, Wendy Stone, Nicolette Adams, Rob Smith, Murray Grant, Isabelle Carré, Laura C. Roden, Katherine J. Denby

**Affiliations:** ^1^ Department of Molecular and Cell BiologyUniversity of Cape TownRondebosch7701South Africa; ^2^ School of Life SciencesUniversity of WarwickCoventryCV4 7ALUK; ^3^ Warwick Systems Biology CentreUniversity of WarwickCoventryCV4 7ALUK; ^4^ College of Life and Environmental SciencesUniversity of ExeterExeterEX4 4QDUK

**Keywords:** disease resistance, plant–pathogen interaction, circadian clock, *Botrytis cinerea*, *Arabidopsis thaliana*, defence response, jasmonate, defence gene expression

## Abstract

The circadian clock, an internal time‐keeping mechanism, allows plants to anticipate regular changes in the environment, such as light and dark, and biotic challenges such as pathogens and herbivores. Here, we demonstrate that the plant circadian clock influences susceptibility to the necrotrophic fungal pathogen, *Botrytis cinerea*. Arabidopsis plants show differential susceptibility to *B. cinerea* depending on the time of day of inoculation. Decreased susceptibility after inoculation at dawn compared with night persists under constant light conditions and is disrupted in dysfunctional clock mutants, demonstrating the role of the plant clock in driving time‐of‐day susceptibility to *B. cinerea*. The decreased susceptibility to *B*. *cinerea* following inoculation at subjective dawn was associated with faster transcriptional reprogramming of the defence response with gating of infection‐responsive genes apparent. Direct target genes of core clock regulators were enriched among the transcription factors that responded more rapidly to infection at subjective dawn than subjective night, suggesting an influence of the clock on the defence‐signalling network. In addition, jasmonate signalling plays a crucial role in the rhythmic susceptibility of Arabidopsis to *B. cinerea* with the enhanced susceptibility to this pathogen at subjective night lost in a *jaz6* mutant.

## Significance Statement

The circadian clock impacts plant metabolism and responses to the environment, including host susceptibility to pathogens. Here we demonstrate that Arabidopsis is less susceptible to fungal inoculation at dawn, because the defence transcriptional network is more rapidly activated. Genes differentially expressed at dawn and night were highly enriched for known direct targets of core clock transcription factors, suggesting direct links between the clock and the defence regulatory network.

## Introduction

Plants possess a robust multi‐layered innate immune system to respond to attack by pathogens in their environment. The first line of defence, pathogen associated molecular pattern (PAMP)‐triggered immunity (PTI) is activated following the detection of PAMPs such as flagellin or chitin by pattern recognition receptors at the plasma membrane (Jones and Dangl, 2006; Schwessinger and Zipfel, 2008). Pathogens in turn have evolved effectors which serve both to suppress PTI and secure nutrients and water from the plant host (Macho and Zipfel, 2015). Direct or indirect detection of these effectors by plant resistance (R) proteins activates the second innate immune layer in plants, known as effector‐triggered immunity (ETI) (Dangl and Jones, 2001). Finally, systemic acquired resistance, whereby infection of one part of a plant leads to increased resistance of uninfected tissues to subsequent pathogen challenge forms the final layer of innate immunity in plants (Fu and Dong, 2013). All three branches of innate immunity rely on large scale transcriptional reprogramming in the host plant, which is activated via a complex signalling network that is strongly influenced by the mutual antagonism and co‐operation between jasmonic acid (JA), ethylene (ET) and salicylic acid (SA) signalling pathways (Robert‐Seilaniantz *et al*., 2011).

*Botrytis cinerea* is an ascomycete necrotrophic plant pathogen with a broad host range (Williamson *et al*., 2007). Plants are infected mainly through conidiospores which are released from infected material when disturbed, or by water splash (Choquer *et al*., 2007; Williamson *et al*., 2007). *B*. *cinerea* conidiospores adhere to and germinate on plant surfaces, forming germ tubes and appressorium‐like structures within 12 h of inoculation, prior to penetration and colonisation of the host (Gwynne‐Vaughan, 1922; van Kan, 2006; Choquer *et al*., 2007). Infectious hyphae penetrate the host tissue after enzymatic degradation of cell walls and production of H_2_O_2_, rather than by osmotic pressure, due to the lack of a cell wall between the appressorium and germ tube (van Kan, 2006; Choquer *et al*., 2007). Successful infection of the plant host by *B. cinerea* is achieved through the release of non‐specific phytotoxins, modification of host redox status, suppression of phytoalexin production, and subversion of programmed cell death (van Baarlen *et al*., 2007). The chronology of the transcriptional response of Arabidopsis to *B. cinerea* has been well characterized in a fine‐scale transcriptome profiling experiment, which identified groups of genes that are activated or repressed at the various stages of infection in the first 48 h after inoculation (Windram *et al*., 2012).

Plant defences may be temporally regulated such that they are strongest at the time of maximal vulnerability to infection, e.g. against bacterial pathogens when stomata are open (Zhang *et al*., 2013), or to coincide with the time of day when a pathogen is most abundant, or conditions are most favourable for pathogen growth. Fungal spore release is generally diurnally regulated; ascospores of *Calonectria nivalis* and *Gibberella zeae* (Sanderson, 1970; Paulitz, 1996), and *Leptosphaeria maculans* (Guo and Fernando, 2005) are released nocturnally, usually peaking around 22:00 to 24:00 h. Hartill (1980) found that ascospores of *Sclerotinia sclerotiorum* and conidiospores of *B. cinerea* were most numerous during the morning to early afternoon. The circadian clock is an endogenous time‐keeping mechanism that synchronizes biological processes with the external environment, such that they occur at optimal times of the day (Dodd *et al*., 2005). The plant circadian clock consists of a series of interlocked transcription‐translation feedback loops, with negative feedback between the morning expressed transcription factors CIRCADIAN CLOCK ASSOCIATED 1 (CCA1) and LATE ELONGATED HYPOCOTYL (LHY) and the evening expressed TIMING OF CAB 1 (TOC1), LUX ARHHYTHMO (LUX), EARLY FLOWERING (ELF) 3 and 4 forming the core of the clock (Hsu and Harmer, 2014). While the clock has long been known to allow plants to anticipate predictable daily changes in abiotic stimuli, such as light, only recently has it become apparent that it also allows plants to anticipate interactions with other organisms. It has been demonstrated that defence responses of Arabidopsis to both oomycete and bacterial pathogens vary with the time of day at which infection occurs (Bhardwaj *et al*., 2011; Wang *et al*., 2011; Zhang *et al*., 2013). Temporal variation in susceptibility to *Pseudomonas syringae* pv *tomato* persists in Arabidopsis under constant light conditions, and is abolished in arrhythmic clock mutants (Bhardwaj *et al*., 2011), strongly implicating the circadian clock in modulation of the host immune response.

It was recently reported that *B. cinerea* virulence is regulated by its circadian oscillator and exhibits minimal pathogenicity at dawn (Hevia *et al*., 2015). Here, we show that plant responses to this necrotrophic fungus are also temporally regulated and play a key role in determining the outcome of infection. The plant circadian clock drives variation in susceptibility to *B. cinerea* and we hypothesised that this is achieved through the regulation of expression of key transcription factors (TFs) in the defence network, resulting in time of inoculation‐dependent variation in signalling. We profiled the transcriptome of Arabidopsis leaves inoculated with *B. cinerea* at different times of day and identified a subset of TFs that are known to be direct targets of clock proteins and respond differentially to this pathogen depending on the time at which inoculation occurs. Furthermore, we show that JA/ET‐responsive defence gene expression occurs more rapidly at dawn than night in response to *B. cinerea*, and identify the JA ZIM‐domain protein JAZ6 as a potential link between the circadian clock and JA‐mediated defence in Arabidopsis.

## Results

### Susceptibility of Arabidopsis to *Botrytis cinerea* varies with time of inoculation under diurnal conditions

**Figure 1:**
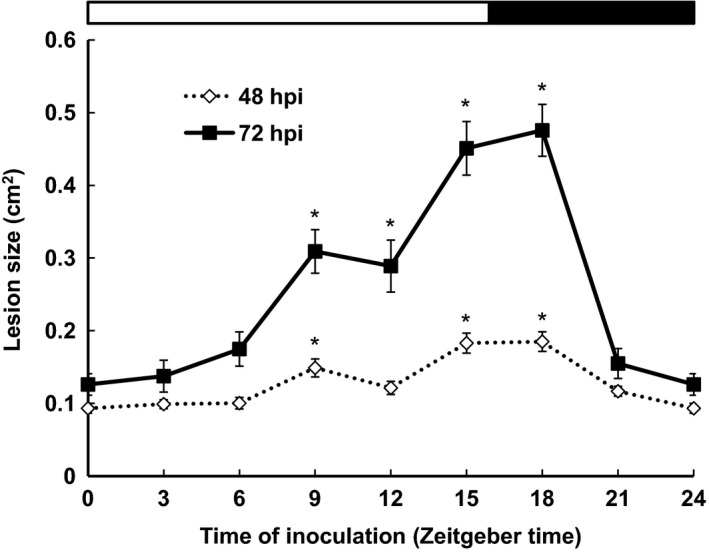
Arabidopsis displays temporal variation in susceptibility to *Botrytis cinerea* under diurnal conditions. Detached leaves from 4‐week‐old plants were inoculated with *B. cinerea* spores at dawn (ZT0) and every 3 h for 24 h under diurnal conditions, and lesion size measured at 48 and 72 h post inoculation (hpi). Data shown are mean values ± standard error of the mean (SEM) (*n *=* *15) from one experiment representative of three. anova revealed a significant effect of time of inoculation on lesion size (*P *< 0.001). Mean lesion sizes significantly different (*P *< 0.01) from those at ZT0 (as determined by Fisher LSD *post‐hoc* analysis) are indicated by *.

To determine whether time of inoculation affects the susceptibility of Arabidopsis to *B. cinerea* under diurnal growth conditions, we inoculated leaves from Col‐0 plants with *B. cinerea* spores every 3 h over a 24 h period under 16 h light/8 h dark (LD) conditions. anova revealed that susceptibility to *B. cinerea* showed significant variation with time of inoculation (*P *<* *0.001), with the smallest lesion size observed after inoculation at dawn (ZT0). Susceptibility to *B. cinerea* increased following inoculations over the course of the day, and peaked after inoculation 2 h after dark (ZT18). A significant difference in lesion size on leaves inoculated at ZT0 versus ZT18 was observed at both 48 and 72 hpi (Figure 1). It should be noted that time of inoculation does not correspond to time of infection in these experiments, as spore germination and penetration of the host tissue by fungal hyphae must first occur. This process takes approximately 10–12 h from the time of inoculation, prior to which no significant differences in host gene expression are observed (Windram *et al*., 2012).

### Temporal variation in susceptibility to *B. cinerea* is driven by the circadian clock

**Figure 2:**
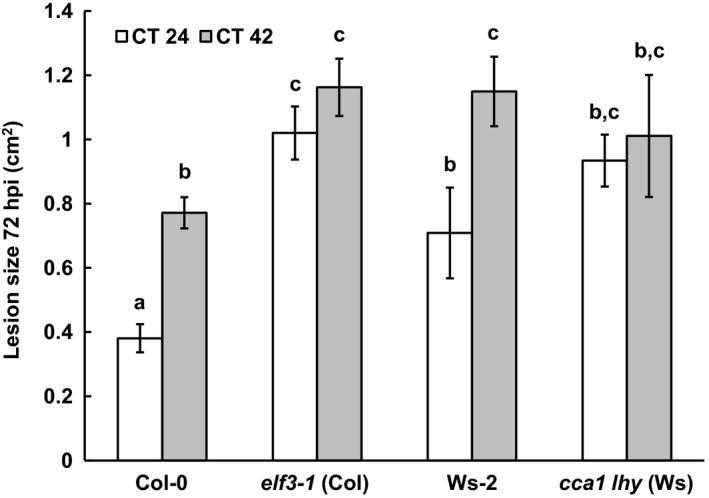
Arabidopsis displays clock‐modulated susceptibility to *Botrytis cinerea*. Detached leaves from 4‐week‐old plants were inoculated with *B. cinerea* spores at CT24 (subjective dawn, white bars) or CT42 (subjective night, grey bars) under LL conditions, and lesion size measured at 72 hpi. Data shown are mean values ± standard error of the mean (SEM) from six (Col‐0 and *elf3‐1*) or three (Ws‐2 and *cca1 lhy*) independent experiments. Twenty leaves were inoculated per genotype/time of inoculation combination in each experiment. anova revealed a significant effect of both host genotype (*P *< 0.001) and time of inoculation (*P *< 0.001) on lesion size. ^a–c^Mean lesion sizes with different letters are significantly different (*P *< 0.01) as determined by Fisher LSD *post‐hoc* analysis.

To determine whether the temporal variation in susceptibility to *B. cinerea* is driven by the plant circadian clock we next inoculated wild‐type plants from two ecotypes (Col‐0 and Ws‐2) with *B. cinerea* spores under free‐running conditions of constant light (LL). Plants were grown for 4 weeks under standard LD conditions, and transferred to LL 24 h prior to inoculation. Detached leaves were inoculated at the time points that Col‐0 plants displayed the greatest resistance and susceptibility under LD conditions, i.e. subjective dawn (corresponding to circadian time 24, CT24) and subjective night (CT42). Under LL conditions, both Col‐0 and Ws‐2 leaves inoculated at CT24 displayed significantly smaller lesions 72 hpi than those inoculated at CT42 (Figure 2), consistent with plant clock‐driven modulation of susceptibility to *B. cinerea*.

If the plant clock is responsible for the rhythmic variation in susceptibility to *B. cinerea* in Arabidopsis, then plants with dysfunctional clocks should display altered patterns of susceptibility to this pathogen, as previously reported for the bacterial pathogen *Pseudomonas syringae* (Bhardwaj *et al*., 2011; Zhang *et al*., 2013). We therefore examined the susceptibility to *B. cinerea* at different times of the day in two mutants with compromised clock function; *elf3‐1* (in the Col‐0 background) which is arrhythmic in LL, and the *cca1 lhy* double mutant (in the Ws‐2 background), which has reduced amplitude, short period rhythms in LL (Mizoguchi *et al*., 2002; Thines and Harmon, 2010). While the *elf3‐1* mutant was more susceptible to *B. cinerea* than Col‐0, there was no temporal variation in lesion size in this line (Figure 2). No significant difference in lesion size between leaves inoculated at CT24 versus CT42 was observed for the *cca1 lhy* double mutant either (Figure 2). This may be the result of its dramatically shortened 18 h period meaning that inoculations at CT24 and CT42 occur by chance at equivalent phases of the circadian cycle. These data provide further evidence that temporal variation in susceptibility to *B. cinerea* in Arabidopsis is driven by a functional circadian clock network.

### *B. cinerea* growth is restricted following inoculation at subjective dawn under LL conditions

The significantly smaller lesion sizes observed after inoculation at CT24 versus CT42 in both Col‐0 and Ws‐2 ecotypes suggest that Arabidopsis is better able to restrict the growth of *B. cinerea* when inoculated at dawn than early night. To better quantify pathogen growth, we examined mRNA levels of the *B. cinerea* tubulin gene 72 hpi in Col‐0 leaf tissue inoculated at CT24 and CT42 under LL conditions using quantitative PCR. We observed that tubulin mRNA levels, relative to those of the host gene *PUX1* (At3 g27310), whose expression is unaffected by *B. cinerea* inoculation or diurnal rhythms (Windram *et al*., 2012), were significantly lower in plants inoculated at CT24 than at CT42 (Figure S1). Plants inoculated at subjective dawn thus appear to mount a more effective defence response to *B. cinerea* than those inoculated at subjective night.

### Inoculation at dawn reduces the chance of a successful infection

**Figure 3:**
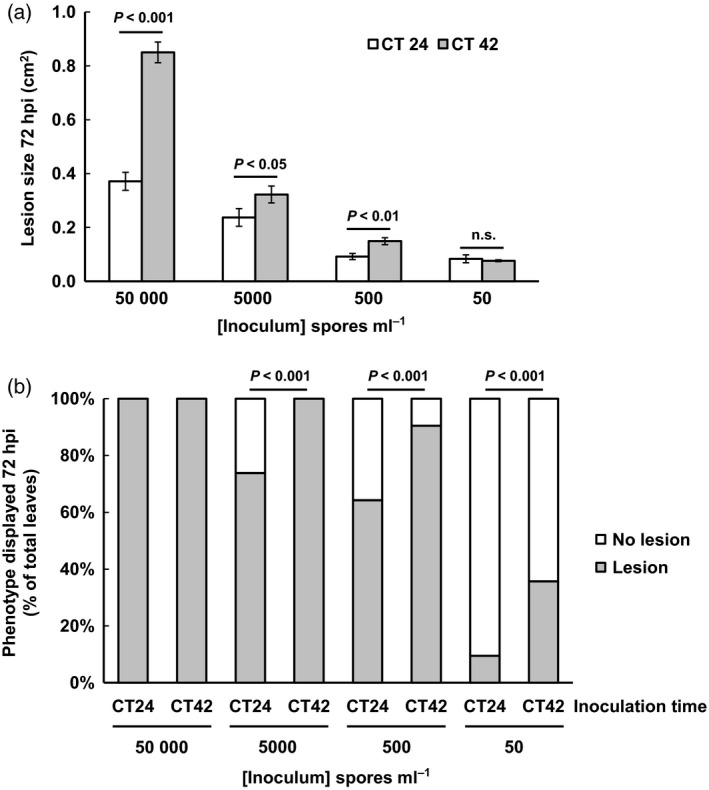
Inoculation at dawn reduces the chance of a successful infection. Detached leaves from 4‐week‐old Col‐0 plants were inoculated with *B. cinerea* spores (ranging from 50 to 50 000 spores mL ^−1^) and disease symptoms assayed at 72 hpi. (a) Mean lesion size ± standard error of the mean (SEM) on leaves that developed disease symptoms (data pooled from three independent experiments, from left to right *n *=* *42, 42, 29, 40, 25, 26, 4 and 15 respectively), *P*‐values from anova for each comparison are indicated. (b) Proportion of leaves displaying disease symptoms (determined as formation of primary lesion) as percentage of total number of leaves inoculated across the three experiments (*n *=* *42). The *P*‐values shown are from chi‐squared analysis testing for a significant relationship between time of inoculation and success of infection at each spore concentration.

Although lesion size was significantly smaller in both Col‐0 and Ws‐2 ecotypes when inoculated at subjective dawn versus subjective night (Figure 2), all leaves inoculated with the standard concentration of *B. cinerea* spores used in our experiments (50 000 spores mL^−1^) developed lesions, indicating that all infections were ultimately successful. We therefore attempted to determine whether clock‐modulation of the defence response might confer a fitness advantage at lower spore inocula concentrations by preventing the development of disease symptoms. As expected, a reduction in lesion area 72 hpi was observed as spore concentration of the inoculum was reduced under LL conditions, though temporal variation in susceptibility was still observed at all spore concentrations tested except 50 spores mL^−1^ (Figure 3a). However, as we observed that not all leaves developed lesions at spore concentrations below 50 000 mL^−1^, we also scored for the presence or absence of lesion formation at 72 hpi. The frequency of successful infections decreased with reducing spore concentration, but was consistently higher on leaves inoculated at CT42 (Figure 3b). Chi‐squared analysis revealed a highly significant relationship between time of inoculation and the frequency of disease symptoms at all three lower spore concentrations (Figure 3b) again indicating that a more effective defence response is launched after inoculation at dawn.

### The host transcriptional response to *B. cinerea* varies with time of inoculation

Large‐scale transcriptional reprogramming following pathogen detection is a major component of the host immune response, with differential expression of approximately one‐third of the Arabidopsis genome occurring within 48 h of inoculation with *B. cinerea* 6 h after dawn (Windram *et al*., 2012). One possible explanation for the temporal variation in susceptibility to *B. cinerea* in Arabidopsis is that the magnitude and/or speed of the transcriptional defence response to this pathogen varies over a 24 h period. To test this hypothesis, we analysed global gene expression using NimbleGen arrays following inoculation of Arabidopsis leaves under LL conditions at either subjective dawn (CT24) or night (CT42). Based on recent high‐resolution expression profiling of the Arabidopsis transcriptome response to *B. cinerea*, we selected 18 and 22 hpi for gene expression analysis as these were time points at which a large proportion of genes first showed differential expression (Windram *et al*., 2012).

As an initial validation of the microarray data, we analysed the expression of 31 marker genes consistently upregulated in response to *B. cinerea* infection in previous studies (Dataset S1). Of these 31 genes, 27 showed a ≥1.5‐fold (log_2_0.6) up‐regulation in response to *B. cinerea* in at least one of the two time points after inoculation at subjective dawn or subjective night, indicating that the infections had triggered the expected transcriptional response in the host. The fact that these studies were carried out on different platforms, and the current study was limited to two time points after inoculation may explain why the remaining four genes were not upregulated as expected.

From the expression data, we identified 901 genes that are responding differentially to inoculation with *B. cinerea* at dawn compared with night (Dataset S2). All 901 differentially expressed genes (DEGs) are significantly up‐ or downregulated in response to *B. cinerea*, but in addition show a significant difference in expression in leaves inoculated at subjective dawn compared with leaves inoculated at subjective night, and/or a significant difference in their response to infection (i.e. fold change in infected versus mock inoculated leaves) at subjective dawn compared to night (see Figure S2).

### Transcription factor genes show both rhythmic basal expression and differential sensitivity to *B. cinerea* infection

Members of a number of TF families, including WRKY, AP2/ERF, NAC and MYB, have been shown to play a role in defence against *B. cinerea* (Birkenbihl and Somssich, 2011). Furthermore, TFs form the backbone of the gene regulatory network mediating transcriptional reprogramming in response to *B. cinerea* infection (Windram *et al*., 2012). As such, temporal variation in TF expression could have a significant impact on the host defence response. Of the 901 genes that show differential expression after inoculation at different times of the day, 99 encode TFs (Dataset S3). These differentially expressed TFs include the known positive regulators of *B. cinerea* defence responses, MYB108 (Mengiste *et al*., 2003) and ERF6 (Moffat *et al*., 2012). Up‐regulation of both TFs was greater at 18 hpi in leaves inoculated at subjective dawn compared with night, indicative of a faster activation of the defence regulatory network following inoculation at dawn.

**Figure 4:**
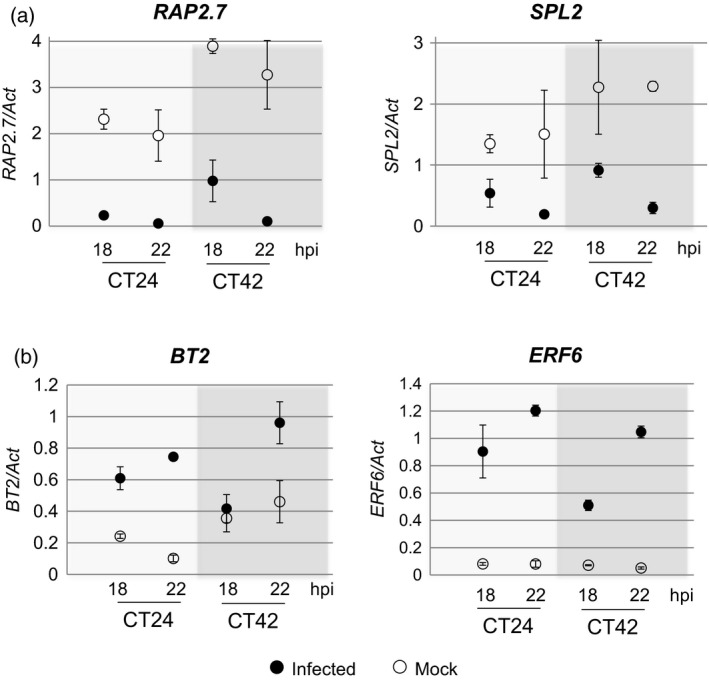
Differential expression (as determined by qPCR) of transcription factor (TF) encoding genes in response to infection at subjective dawn and night under LL conditions. (a) TF genes showing different basal levels of expression at CT24 (subjective dawn) or CT42 (subjective night), but a similar fold change in response to infection and (b) TF genes with a different fold change in response to infection at CT24 or CT42. Filled circles are expression values after *B. cinerea* infection and open circles from mock inoculated leaves. Values shown are mean expression values (normalized to *Actin2* expression) from three biological replicates ± standard error of the mean (SEM).

We investigated whether the more rapid activation of the defence network was a result of differential basal expression of key TFs such that the same fold change in expression following infection results in significantly different absolute expression levels (expression level‐only genes in Dataset S3 and Figure S2b) or whether there was differential induction/repression of TF genes after inoculation at subjective dawn versus subjective night leading to different expression levels (both a fold change and expression level difference in Dataset S3 and Figure S2c). This latter situation can be interpreted as gating of the defence response by the clock so that the magnitude of the host response to pathogen detection varies with time of day. We found evidence for both types of regulation in up‐ and downregulated TF genes (Figure S3). We also found TFs that had a different fold change in response to infection at subjective dawn or night but the resulting expression levels were not significantly different (fold change only in Dataset S3). This suggests that the level of expression of these genes is tightly controlled and/or there is a maximal level of expression for these genes. We selected four of the differentially expressed TFs for validation of their expression profiles using qPCR. The qPCR analysis, also carried out on samples from plants inoculated under LL conditions, confirmed the differential expression of these TFs after inoculation at subjective dawn or night (Figure 4). Enhanced expression of TF genes after inoculation at dawn appears to be driving a more rapid host defence response, potentially leading to the observed decrease in susceptibility to *B. cinerea* infection.

Approximately half of the TFs differentially expressed after inoculation at subjective dawn or night show circadian expression patterns in the data sets analysed by Hazen *et al*. (2009) or Covington *et al*. (2008), which may drive differences in basal expression in these genes. Strikingly, the differentially expressed TFs are highly enriched for genes that are direct targets of the core clock components (Dataset S3). Direct targets of TOC1 (Huang *et al*., 2012), PRR5 (Nakamichi *et al*., 2012), and PRR7 (Liu *et al*., 2013) were significantly enriched (*P *< 0.0001) compared with the genome as a whole indicating a high level of input from the clock on these defence‐related regulators.

### Hormone responsive genes are differentially expressed after inoculation at dawn or night

To gain insight on the biological processes represented by the genes differentially responding to inoculation at dawn or night, we grouped the 901 DEGs into eight groups on the basis of their expression patterns (Dataset S2). The first four groups are: (i) UPUP18 – genes upregulated in response to infection and with a higher expression level and/or induction after inoculation at dawn compared to night by 18 hpi; (ii) UPUP22 – genes upregulated in response to infection and with a higher expression level and/or induction after inoculation at dawn compared to night only at 22 hpi; (iii) and (iv) the corresponding downregulated groups, DOWNDOWN18 (genes downregulated in response to infection and with a lower expression level and/or greater repression after inoculation at dawn compared to night by 18 hpi) and DOWNDOWN22 (lower expression and/or greater repression after inoculation at dawn compared with night only observed at 22 hpi). These four groups contained the majority (71%) of the DEGs: UPUP18 – 219 DEGs, UPUP22 – 44 DEGs, DOWNDOWN18 – 257 DEGs, DOWNDOWN22 – 123 DEGs. These appear to be genes whose response to infection is amplified or occurs more rapidly after inoculation at dawn compared to night and hence may underlie the enhanced defence response after inoculation at dawn.

The remaining 246 DEGs showed a less intuitive expression pattern. Forty‐seven and 98 DEGs were up‐regulated in response to infection, but showed lower expression or reduced response to infection after inoculation at subjective dawn compared with night at 18 or 22 hpi respectively (UPDOWN18, UPDOWN22). Similarly 53 and 48 DEGs were downregulated in response to infection but showed higher expression or response to infection after inoculation at subjective dawn compared to night at 18 and 22 hpi respectively (DOWNUP18, DOWNUP22).

**Figure 5:**
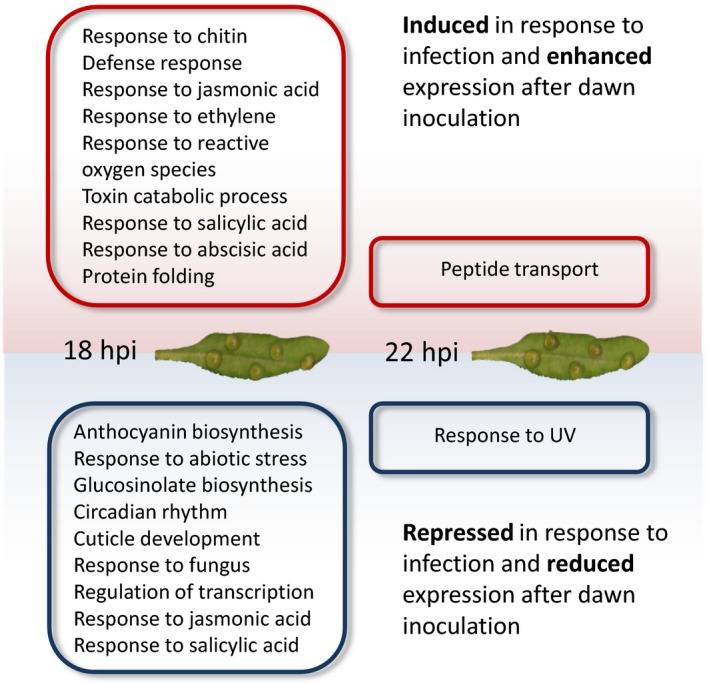
Biological processes associated with the enhanced defence response at subjective dawn. The figure indicates selected Gene Ontology (GO) biological process terms enriched in genes whose response to infection is enhanced at subjective dawn compared with subjective night.

We focused on the four main groups of genes with enhanced response to inoculation at subjective dawn and investigated the biological processes these genes are involved in using enrichment of Gene Ontology (GO) terms (Ashburner *et al*., 2000) (Figure 5 and Dataset S4). This analysis suggested that the majority of functionally coordinated changes in gene expression occur by 18 hpi; very few GO terms were enriched in genes only showing a difference in expression between subjective dawn and night inoculation at 22 hpi. The presence of the GO term ‘circadian rhythm’ in the genes repressed in response to infection is expected and an initial validation of our expression profiling approach. We know that the amplitude of core clock gene expression is reduced in response to *B. cinerea* infection (Windram *et al*., 2012) and that these genes are obviously expressed at different basal levels at dawn and night.

Several of the terms in Figure 5 (including toxin catabolism and glucosinolate biosynthesis) match those identified in our previous expression time series following inoculation of Arabidopsis leaves with *B. cinerea* 6 h after dawn (Windram *et al*., 2012) suggesting that a specific sector of the defence response is temporally regulated. A striking observation is the abundance of terms associated with hormone signalling and response pathways both in the groups induced and repressed in response to infection, as well as terms directly related to the perception and response to fungal infection. Notably, genes responding to four phytohormones (ET, JA, SA and ABA) are enriched in upregulated genes showing an enhanced response to infection by 18 hpi at dawn compared to night (UPUP18).

**Figure 6:**
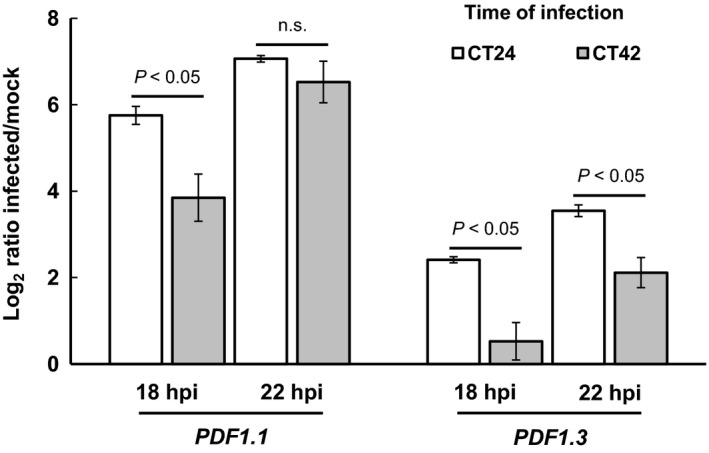
Differential induction of *PDF* gene expression in response to inoculation at subjective dawn versus night. Detached leaves were inoculated with *B. cinerea* spores or mock inoculated at CT24 or CT42 under LL conditions, and harvested at 18 and 22 hpi for RNA extraction. Relative expression of *PDF1.1* and *PDF1.3* was determined by qPCR (with normalization to *Actin2* levels) and log_2_ expression ratios calculated by normalizing *PDF*/*Act2* values from infected leaves to those in mock inoculated controls for each time point. Values are mean log_2_ expression ratios (± SEM) from three biological repeats, and *P*‐values shown are from one‐way anova testing for a significant difference in induction between leaves inoculated at CT24 versus CT42. n.s., not significant.

We used the meme‐lab software (Brown *et al*., 2013) to determine whether the promoters of genes responding differentially to infection at different times of the day were enriched for particular DNA sequences. The meme‐lab results are given in Dataset S5 but of particular interest was a motif enriched in the promoters of genes in the UPUP18 group. This motif, a GCC‐box motif, was found in the promoters of five genes, three of which were plant defensins (*PDF1.2*,* 1.2b* and *1.2c*). The GCC‐box has been identified as a binding site for a number of AP2/Ethylene response factor (ERF) TFs and synergistic JA/ET signalling is known to converge on the GCC‐box (Zarei *et al*., 2011). Two other plant defensins (*PDF1.1* and *PDF1.3*) were also in the UPUP18 group and using the position weight matrix for the GCC‐box found by meme‐lab, a motif similar to the GCC‐box was identified in the upstream promoter sequence of *PDF1.3* (ATCATCAGCCCA). We analysed the expression of *PDF1.1* and *PDF1.3* following inoculation with *B. cinerea* at subjective dawn and night using qPCR (Figure 6). As expected, both genes were induced in response to *B. cinerea* infection and, as seen in our array data, their expression was significantly higher at 18 hpi (and for *PDF1.3* also at 22 hpi) in plants inoculated at subjective dawn compared with subjective night (Figure 6). These results suggest that activation of JA/ET‐mediated defence gene expression is indeed more rapid in plants inoculated at dawn.

### Jasmonate signalling is required for circadian‐driven variation in *B. cinerea* susceptibility

**Figure 7:**
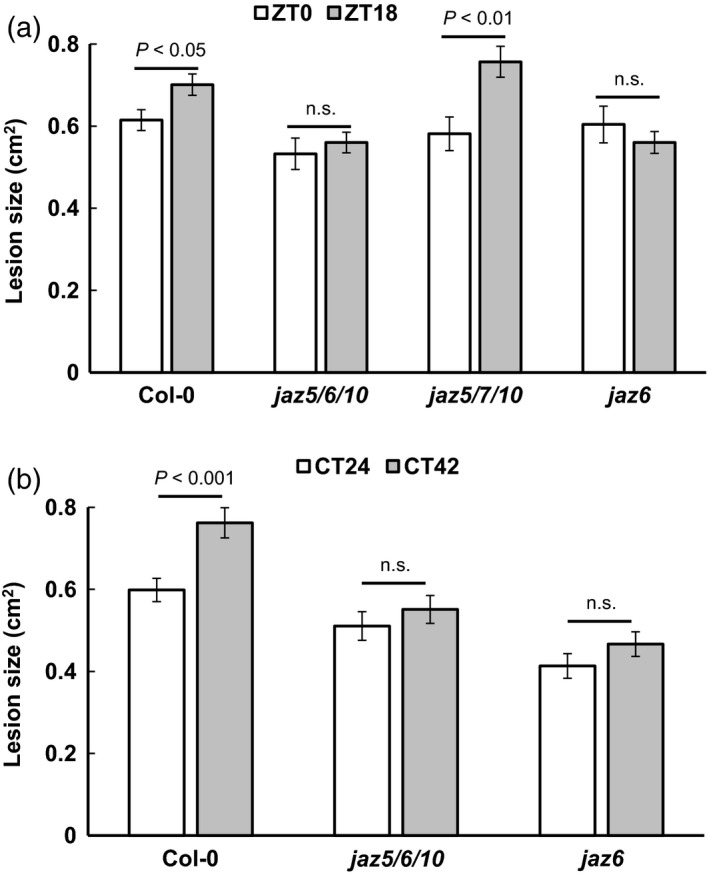
Temporal variation in susceptibility to *B. cinerea* requires JAZ6. Detached leaves from 4‐week‐old plants were inoculated with *B. cinerea* spores under: (a) LD at dawn or night (ZT0 and ZT18) or (b) LL at subjective dawn or night (CT24 and CT42) conditions and lesion area measured at 72 hpi. Data shown are mean lesion sizes ± standard error of the mean (SEM) on wild‐type (Col‐0) leaves versus *jaz* mutants (*n* ≥ 30 for each time of inoculation/genotype combination). anova was used to test whether lesion size was significantly different between leaves inoculated at dawn versus night; *P*‐values for each comparison are shown. n.s., not significant.

Jasmonate ZIM‐domain (JAZ) proteins are transcriptional repressors that act as negative regulators of JA and ET‐mediated defence responses, through their interaction with COI1, and the MYC2/3/4 and EIN3/EIL1 TFs (Chini *et al*., 2007; Fernández‐Calvo *et al*., 2011; Zhu *et al*., 2011). Upon perception of the JA‐Ile conjugate, COI1 interacts with JAZs targeting them for degradation, thereby relieving their repressive effect on MYC and EIN3/EIL1‐mediated gene expression (Thines *et al*., 2007). To determine whether JA signalling might play a role in clock‐modulated susceptibility to *B. cinerea*, we analysed lesion formation in *jaz* mutants, reasoning that JAZ‐mediated repression of JA signalling would be compromised in these plants. Because of potential redundancy in JAZ function, we began by analysing lesion formation in *jaz* triple mutants in which redundancy may be overcome. In contrast to Col‐0, temporal variation in susceptibility to *B. cinerea* was not observed in the *jaz5*,*6*,*10* triple mutant under LD conditions (Figure 7a). However, *jaz5*,*7*,*10* displayed the normal enhanced susceptibility after inoculation at night. The difference between these two triple mutants suggested that *JAZ6* could be a key player in circadian regulation of defence against *B. cinerea*. Furthermore, the expression of the *JAZ6* gene is markedly induced in response to *B. cinerea* infection (Figure S4). Consistent with this hypothesis, the single *jaz6* mutant did not show a significant time‐of‐day difference in lesion size after inoculation at dawn or night (Figure 7a). This lack of temporal variation in susceptibility in the *jaz6* and triple *jaz5*,*6*,*10* mutants was also evident under LL conditions (Figure 7b). These results indicate that JAZ6 is required for circadian‐driven variation in susceptibility to *B. cinerea* and may be responsible for repression of JA/ET‐responsive genes after inoculation at subjective night.

## Discussion

### The plant circadian clock influences the outcome of *Botrytis cinerea* infections

We have demonstrated that Arabidopsis infection by the necrotrophic pathogen *B. cinerea* leads to different outcomes, depending on the time of the day at which plants are inoculated with fungal spores. Under diurnal (LD) conditions lowest susceptibility to *B. cinerea* was observed in leaves inoculated at dawn (ZT0) (Figure 1). Thereafter susceptibility to this pathogen increased during the light period, and peaked when leaves were inoculated 18 h after dawn, 2 h into the dark period (ZT18). This variation in host susceptibility could result from differences in pathogen virulence and/or the host defence response over the course of the day.

Light has previously been reported to negatively affect the virulence of *B. cinerea*, with reduced lesion sizes reported on Arabidopsis leaves infected under LL versus LD conditions (Canessa *et al*., 2013). However, we found differences in lesion sizes between inoculations carried out at different times during the light period, for example ZT0 versus ZT9, 12 or 15, as well as the dark period, ZT18 versus ZT21; whereas lesion sizes following inoculations at ZT0 or ZT3 in the light were not significantly different from those at ZT21 in the dark (Figure 1). These results indicate that the time‐of‐day variation in the success of *B. cinerea* infection is not due simply to the presence or absence of light. A recent study demonstrated that the *B. cinerea* circadian clock contributes to the outcome of infection, and the pathogen was shown to have maximal virulence to Arabidopsis plants when inoculated at dusk (Hevia *et al*., 2015). Although conidiation is promoted by light in a number of *B. cinerea* strains (Canessa *et al*., 2013), the *B. cinerea* pepper isolate used in our experiments produces conidia irrespective of the presence of light. Crucially, in our experiments the pathogen was cultured in constant darkness, which provided no entrainment for the clock, and was exposed to light only when the conidiospores were removed immediately prior to use in inoculations, after which infections developed in constant light. Thus, the pathogen was in an equivalent state at each inoculation time and unlikely to be the cause of the time‐of‐day variation observed in Figure 1. Instead our observations that the time of inoculation variation in susceptibility to *B. cinerea* persists under constant light (LL) conditions in both Col‐0 and Ws ecotypes, but not in dysfunctional clock mutants in these genetic backgrounds, strongly suggest that temporal variation in resistance to *B. cinerea* infection is mediated by the plant circadian clock. The demonstration that mutation of a single gene, *JAZ6*, causes loss of time‐of‐day variation in susceptibility under both LD and LL conditions again indicates that the outcome of infection is driven by plant mechanisms.

### Comparison of rhythmic defence against biotrophic and necrotrophic pathogens

We have previously shown that Arabidopsis plants pressure‐inoculated with *P. syringae* display maximal resistance to this pathogen in the subjective morning and are most susceptible during the subjective night (Bhardwaj *et al*., 2011). Given the different lifestyles of *B. cinerea* and *P. syringae* (necrotroph and hemi‐biotroph), and the documented antagonism between hormonal defence pathways against these pathogens (Koornneef and Pieterse, 2008), it may seem surprising that a similar temporal pattern of host resistance was observed. However, while infiltration of *P. syringae* directly into the apoplast results in a host transcriptional response within 2 h (Truman *et al*., 2006), no significant differences in host gene expression were observed in Arabidopsis leaves inoculated with *B. cinerea* spores until 10 hpi (Windram *et al*., 2012). This delay is likely because *B. cinerea* conidiospores must germinate in the inoculation droplet and subsequently form the infectious hyphae required for enzymatic degradation of the host cell wall and penetration of the host. Thus plants inoculated with *B. cinerea* spores at CT24 may only respond to the pathogen at around CT34 (late subjective day), and those inoculated at CT42 at around CT52 (subjective morning). If so, then the true times of maximal resistance to *P. syringae* and *B. cinerea* would be 12 h out of phase, mirroring clock‐modulated SA and JA levels which peak during the middle of the subjective night and day respectively in uninfected plants under constant dark conditions (Goodspeed *et al*., 2012). Similarly under LD conditions, genes associated with JA signalling peak 10–12 h after dawn, again coinciding with the first plant responses to *B. cinerea* inoculation at dawn (Windram *et al*., 2012).

### Mechanism of clock‐driven defence

How does the circadian clock influence host susceptibility to *B. cinerea?* Without knowing the biology of *B. cinerea*, an obvious suggestion would be that clock regulation of stomatal aperture (Dodd *et al*., 2005) could influence entry of the pathogen to the host. The opportunistic use of open stomata and wounds has been reported during infection by *B. cinerea* (Fourie and Holz, 1995), although the primary route of entry is via penetration of the host cuticle (Williamson *et al*., 2007). We examined hyphal behaviour following spore germination on Arabidopsis leaves and found no evidence of directional growth of *B. cinerea* hyphae towards the stomata (Figure S5a). Indeed, on several occasions we observed hyphae growing over an open stomate (Figure S5b). Hence, it does not appear that stomata are important sites of entry into Arabidopsis for *B. cinerea*, and the temporal variation in immunity to this pathogen is unlikely to be explained by clock‐driven changes in stomatal aperture.

Our results suggest that the difference in susceptibility of Arabidopsis to *B. cinerea* at different times of the day is mediated by differential activation of the plant defence response with preferential induction of the defence response following inoculation at dawn. We identified a set of genes that exhibited an enhanced response to inoculation at subjective dawn compared with night and are involved in processes previously linked to the plant defence response against *B. cinerea* (Figure 5; Windram *et al*., 2012). Our data suggest that activation of, and/or flow of information through, the defence regulatory network is enhanced after inoculation at subjective dawn compared with at night. Several known key regulators of susceptibility to *B. cinerea* were more rapidly induced or repressed in response to inoculation at subjective dawn than night, including *ERF1* (Berrocal‐Lobo *et al*., 2002); *ERF6* (Moffat *et al*., 2012); *MYB108* (Mengiste *et al*., 2003) and *WRKY60* (Xu *et al*., 2006). TFs responding differentially to inoculation at dawn compared to night were highly enriched for known direct targets of core clock TFs, suggesting direct links between the circadian clock and the defence regulatory network.

### Role of the jasmonate pathway

It is well known that the plant hormone JA influences the outcome of infection by *B. cinerea* (Thomma *et al*., 1998) and it was clear from our transcriptome profiling that it plays a key role in the time‐of‐day variation of susceptibility to *B. cinerea*. JA biosynthesis and signalling are known to be under the control of the circadian clock and clock‐driven accumulation of JA during the day was shown to contribute to protection against grazing by the herbivore *Trichoplusia ni* (Goodspeed *et al*., 2012). Furthermore, the clock component TIME FOR COFFEE (TIC) mediates rhythmic expression of the JA receptor *COI1* under diurnal conditions and temporal gating of at least some JA responses (Shin *et al*., 2012). TIC interacts directly with MYC2 preventing accumulation of this TF (Shin *et al*., 2012) which is also regulated by binding of JAZ repressors (Chini *et al*., 2007).

We have identified JAZ6 as a crucial component in of time‐of‐day defence against *B. cinerea* and suggest that JAZ6 is another important link between the clock and JA signalling pathways. JAZ proteins regulate JA responses by forming a repressive complex with various TFs and the co‐repressor Topless, often via the adapter protein NINJA (Pauwels *et al*., 2010) although JAZ6 contains the repressive EAR domain suggesting it may independently repress transcription. After binding of activated JA (JA‐Ile) to the JA receptor COI1, JAZ proteins are targeted for degradation by the 26S proteasome (Thines *et al*., 2007) relieving repression on the bound TFs thereby resulting in activation of downstream JA processes. We would expect the absence of *JAZ6* to relieve repression of a positive regulator of defence leading to decreased susceptibility at night. Why does JAZ6 only exert its effect at night? *JAZ6* expression is under circadian control (Covington *et al*., 2008; Hazen *et al*., 2009) but its induction in response to *B. cinerea* infection is significantly higher (Figure S4). Future measurement of JAZ6 protein levels, and its presence in repressive defence‐related TF complexes, will indicate whether temporal variation in JAZ6 accumulation or activity is responsible for restricting its effects on the defence response to night.

### Adaptive significance of time‐of‐day variation in defence

We propose two hypotheses to explain the time‐of‐day variation in susceptibility to pathogens in plants. Plants may anticipate an increased likelihood of infection at dawn or circadian regulation of plant processes (such as metabolic pathways) for non‐defence‐related reasons may result in the defence response being sub‐optimal at night. *B. cinerea* may have exploited such a temporal gap in plant defences and used its own circadian clock to align attack strategy with times of plant least resistance (Hevia *et al*., 2015). There is evidence to support the first anticipatory hypothesis. Circadian and diurnal rhythms of spore development or release have been noted in many fungal genera (Ingold, 1971) with these spore release rhythms persisting in some species even in constant laboratory conditions (Pittendrigh *et al*., 1959; Ingold, 1971; Canessa *et al*., 2013) indicating endogenous control. The morning reduction in humidity and increase in temperature causes release of *B. cinerea* conidia into the air (Williamson *et al*., 2007) resulting in greatest spore abundance during the morning to early afternoon (Hartill, 1980). The likelihood of successful infection of Arabidopsis leaves by *B. cinerea* was lower following inoculation at subjective dawn than subjective night (Figure 3b), consistent with the idea that plant defences are enhanced in anticipation of the abundance of spores in the morning. Further understanding of how the plant clock is driving a more effective defence response after inoculation at dawn will help in pinpointing crucial regulatory components that could be used to enhance resistance against this economically important fungal pathogen.

## Experimental Procedures

### Plant material and growth conditions

Arabidopsis seeds were sown on a 1:1 mix of peat (Jiffy Products, Norway, http://www.jiffygroup.com/) and vermiculite and stratified for 48 h at 4°C in the dark. Plants were grown under a long‐day photoperiod (16 h light, 8 h dark) at 22°C and 55% relative humidity, and cool white fluorescent light of 80–100 μmol m^−2^ sec^−1^. Where plants were to be infected with *B. cinerea* under constant light (LL), they were transferred to LL conditions during the 16 h light period 24 h prior to inoculation. The *cca1‐11 lhy‐21* double mutant (N9809) was obtained from the Nottingham Arabidopsis Stock Centre. The *elf3‐1* mutant (Zagotta *et al*., 1992) was a kind gift from Frank Harmon. Single and triple *jaz* mutants are described in de Torres Zabala *et al*. (2015). *JAZ6* mRNA levels 18 hpi with *B. cinerea* were greatly reduced in the *jaz6* mutant relative to those observed in wild‐type plants (Figure S6).

### *Botrytis cinerea* inoculations

*Botrytis cinerea* pepper isolate (described in Denby *et al*., 2004) was sub‐cultured on apricot halves in the dark at 25°C 2 weeks prior to use of the spores. To determine the susceptibility of Arabidopsis to *B. cinerea* at each time of inoculation detached leaves were inoculated with 10 μL of half‐strength grape juice containing 5 × 10^4^ spores mL^−1^, as previously described (Ingle and Roden, 2014). Lesions were photographed 48–72 h post inoculation (hpi), and lesion area determined using imagej (http://rsbweb.nih.gov/ij/). For microarray experiments, leaf five was detached from 4‐week‐old wild‐type (Col‐0) plants, inoculated with six 10 μL droplets of the spore solution (spaced evenly over the leaf surface) at CT24 (subjective dawn) or CT42 (subjective night), and harvested 18 or 22 hpi. For all experiments half‐strength grape juice served as the mock infection control. Lactophenol–trypan blue staining (Koch and Slusarenko, 1990) was used for microscopic visualisation of hyphal structures following conidiospore germination.

### NimbleGen microarray analysis

Total RNA was extracted from four single Arabidopsis leaves using Trizol (Ambion, ThermoFisher Scientific, https://www.thermofisher.com/za/en/home.html), and cleaned up using the RNeasy kit (Qiagen, https://www.qiagen.com/za/) with on‐column DNase digestion. Equal amounts of total RNA were pooled from the four biological replicates for each of the eight experimental treatments (18 hpi at CT24, 22 hpi at CT24, 18 hpi at CT42 or 22 hpi at CT42 with *B. cinerea* spores or half‐strength grape juice) and 100 ng of each pool amplified using the MessageAmp II kit (Life Technologies). Double‐stranded cDNA was synthesised from 2.5 μg of the resulting aRNA using the Superscript Double‐stranded cDNA synthesis kit (Life Technologies). Following RNase A digestion, ds cDNA was purified using Qiaquick columns (Qiagen). Five hundred ng of ds cDNA was labelled using the NimbleGen One‐Color DNA labelling kit (Roche, https://lifescience.roche.com/shop/home), as per the manufacturer's instructions except that half volumes of all reagents were used for all steps. Four μg of the resulting Cy3‐labelled cDNA was hybridised against NimbleGen *A. thaliana* gene expression 12 × 135K arrays custom designed for the TAIR10 *Arabidopsis thaliana* genome annotation (Design ID OID37507), two duplicate arrays per sample, which were washed and scanned using the NimbleGen MS 200 scanner as per the manufacturer's instructions. The RMA algorithm (Irizarry *et al*., 2003) was used to normalize the expression data and generate log_2_ expression values for each gene. All raw and normalized microarray data has been deposited in GEO (GSE70137). A three‐factor anova was performed to identify genes showing significant expression differences due to infection with *B. cinerea* (I), time of day of inoculation (ToD), time after inoculation (T) and/or interaction between these factors, and a Bonferroni multiple testing correction applied. Genes whose expression showed a significant effect of I × ToD, I × ToD × T, or a significant effect of I and ToD, or I and ToD × T were selected and filtered to only keep genes showing a ≥log_2_0.6 change in expression due to *B. cinerea* infection at any time point. Further filtering was done to select genes showing a ≥log_2_0.6 difference in expression between infected samples inoculated at dawn compared with night, and/or a ≥log_2_0.6 difference in the ratio of infected:mock at dawn compared with night.

### Gene Ontology and promoter sequence motif analysis

The DEG were grouped depending on their expression profiles (see Methods S1) and analysed for overrepresented Gene Ontology (Ashburner *et al*., 2000) terms. The same groups were tested for overrepresentation of known TF binding motifs in their upstream promoter sequences as outlined in Breeze *et al*. (2011).

### Quantitative PCR analysis

Quantitative PCR was performed using a RotorGene RG3000A instrument (Corbett Research, Australia, https://www.qiagen.com/za/corbett/welcome.aspx). The cDNA for these experiments was synthesised from 1 μg of total RNA for each independent biological replicate (leaf 5 from a single Col‐0 plant) using Superscript III reverse transcriptase (Life Technologies). The qPCR reactions consisted of 1 μL template cDNA, 5 μL Kapa SYBR FAST Universal 2× qPCR Master Mix (Kapa Biosystems, South Africa, https://www.kapabiosystems.com/), and 200–900 nm of each primer in a final volume of 10 μL. Amplification conditions were as follows; an initial step at 95°C for 3 min, followed by 40 cycles of 95°C for 3 sec, primer annealing at 58 or 60°C for 20 sec and elongation at 72°C for 1 sec. The relative expression level of each gene of interest was calculated with the rotorgene 6000 (Corbett Life Science, https://www.qiagen.com/za/corbett/welcome.aspx) series software v1.7 using the two standard curve method. Details of the primers used and specific qPCR reaction conditions can be found in Table S1. *Actin2* (At3 g18780) and *PUX1* (At3 g27310) were used to normalize expression data in these experiments as mRNA levels of these genes are unaffected by *B. cinerea* infection (Windram *et al*., 2012), and constant across our mock inoculation microarray samples.

## Author Contributions

RAI, LCR, IAC and KJD had the original ideas for the research. RAI, CS, WS, NA, RS and MG carried out experiments. All authors had input into the design of experiments and data analysis. RAI, LCR and KJD wrote the manuscript with contributions from all authors.

## Supporting information


**Figure S1.** Growth of *Botrytis cinerea* is restricted in plants inoculated at subjective dawn versus subjective night.

**Figure S2.** Diagram illustrating selection of differentially expressed genes.

**Figure S3.** Differential expression of transcription factor (TF) encoding genes in response to infection at subjective dawn or night under LL conditions.

**Figure S4.** *JAZ6* expression is transiently induced during *B. cinerea* infection.

**Figure S5.** Stomata are not a primary point of entry for *B. cinerea* hyphae during infection of Arabidopsis.

**Figure S6.** Expression of *JAZ6* in the *jaz6* mutant line and Col‐0 18 hpi with *B. cinerea* or mock control.

**Table S1.** Primers used in quantitative PCR experiments.

**Methods S1.** Grouping of the differentially expressed genes for Gene Ontology and motif analysis.

**Dataset S1.** Expression data 18 and 22 hpi after subjective dawn and night inoculations with *B. cinerea* for genes with consistent expression patterns in previous studies.

**Dataset S2.** Arabidopsis genes whose expression changes significantly in response to *B. cinerea* and to the time of day at which inoculation occurred.

**Dataset S3.** Transcription factor genes that are differentially expressed in response to inoculation with *B. cinerea* at different times of the day.

**Dataset S4.** Biological Process Gene Ontology terms significantly overrepresented in groups of genes differentially expressed in response to inoculation at different times of the day.

**Dataset S5.** Motifs significantly overrepresented in upstream regions of groups of genes differentially expressed in response to inoculation at different times of the day.
