# Supplementary material for: Jasmonate signalling drives time‐of‐day differences in susceptibility of Arabidopsis to the fungal pathogen Botrytis cinerea
Source: Plant J. 2015 Nov 21;84(5):937–48. doi: 10.1111/tpj.13050 (PMC4982060; doi:10.1111/tpj.13050)
Supplement: Supplementary file 1 — Figure S1. Growth of Botrytis cinerea is restricted in plants inoculated at subjective dawn versus subjective night. [file TPJ-84-937-s001.pptx]

## Slide 1
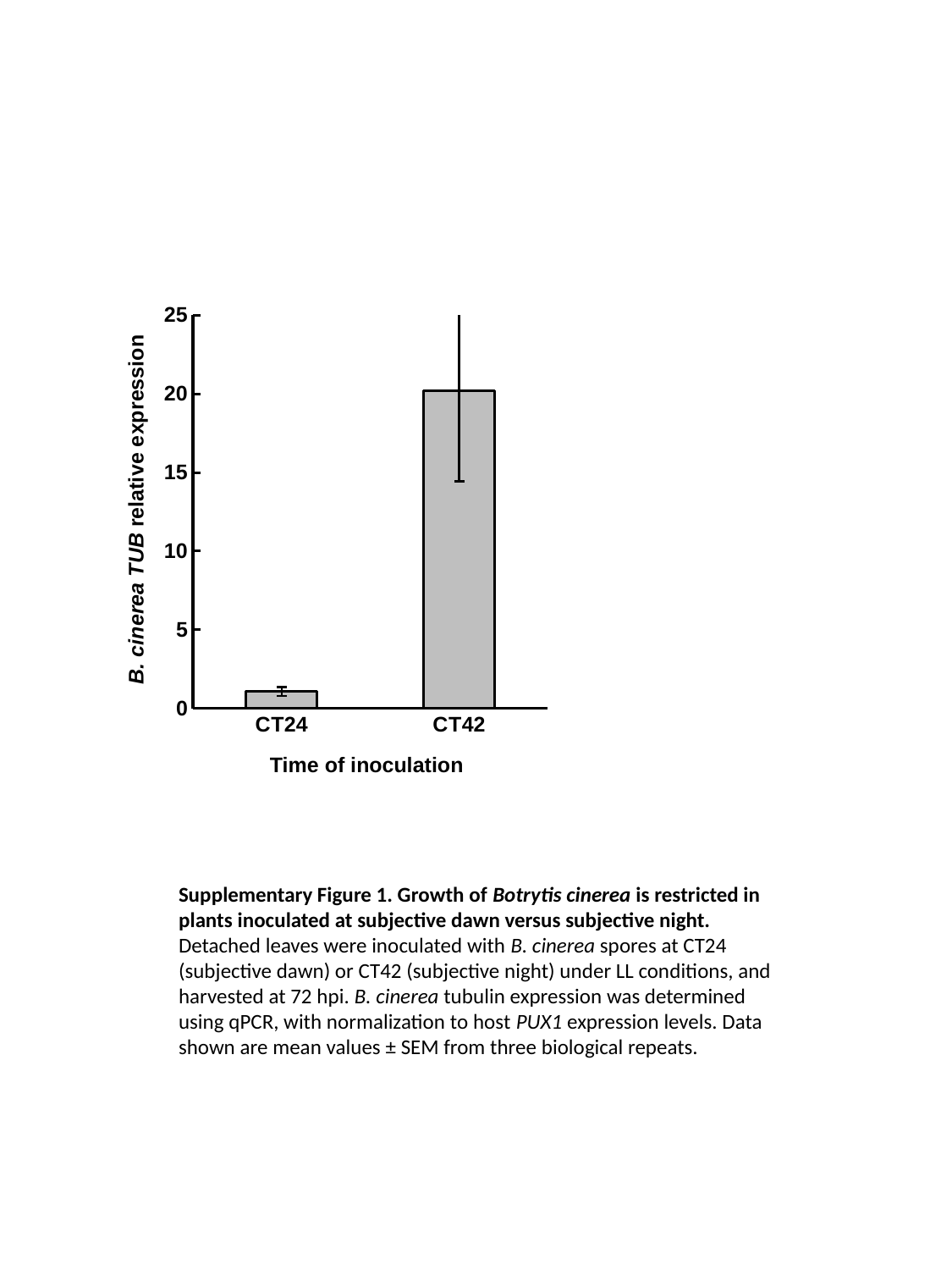

### Chart
| Category | TUB/PUX |
|---|---|
| CT24 | 1.074688917157253 |
| CT42 | 20.20425903740759 |B. cinerea TUB relative expression
Time of inoculation
Supplementary Figure 1. Growth of Botrytis cinerea is restricted in plants inoculated at subjective dawn versus subjective night. Detached leaves were inoculated with B. cinerea spores at CT24 (subjective dawn) or CT42 (subjective night) under LL conditions, and harvested at 72 hpi. B. cinerea tubulin expression was determined using qPCR, with normalization to host PUX1 expression levels. Data shown are mean values ± SEM from three biological repeats.
