# Supplementary material for: Jasmonate signalling drives time‐of‐day differences in susceptibility of Arabidopsis to the fungal pathogen Botrytis cinerea
Source: Plant J. 2015 Nov 21;84(5):937–48. doi: 10.1111/tpj.13050 (PMC4982060; doi:10.1111/tpj.13050)
Supplement: Supplementary file 2 — Figure S2. Diagram illustrating selection of differentially expressed genes. [file TPJ-84-937-s002.pptx]

## Slide 1
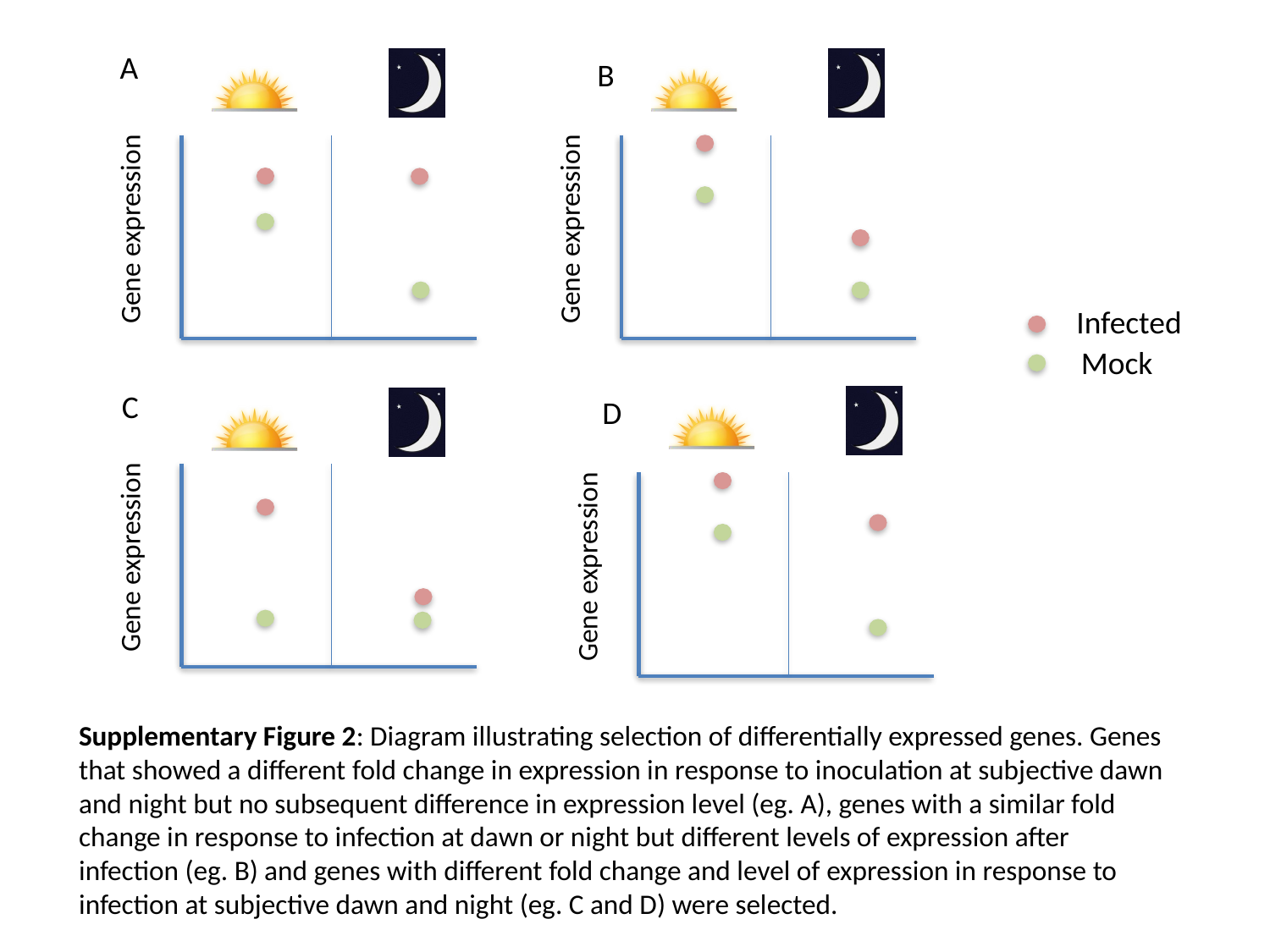

A
Gene expression
Gene expression
B
Infected
Mock
C
Gene expression
D
Gene expression
Supplementary Figure 2: Diagram illustrating selection of differentially expressed genes. Genes that showed a different fold change in expression in response to inoculation at subjective dawn and night but no subsequent difference in expression level (eg. A), genes with a similar fold change in response to infection at dawn or night but different levels of expression after infection (eg. B) and genes with different fold change and level of expression in response to infection at subjective dawn and night (eg. C and D) were selected.
