# Supplementary material for: Jasmonate signalling drives time‐of‐day differences in susceptibility of Arabidopsis to the fungal pathogen Botrytis cinerea
Source: Plant J. 2015 Nov 21;84(5):937–48. doi: 10.1111/tpj.13050 (PMC4982060; doi:10.1111/tpj.13050)
Supplement: Supplementary file 3 — Figure S3. Differential expression of transcription factor (TF) encoding genes in response to infection at subjective dawn or night under LL conditions. [file TPJ-84-937-s003.pptx]

## Slide 1
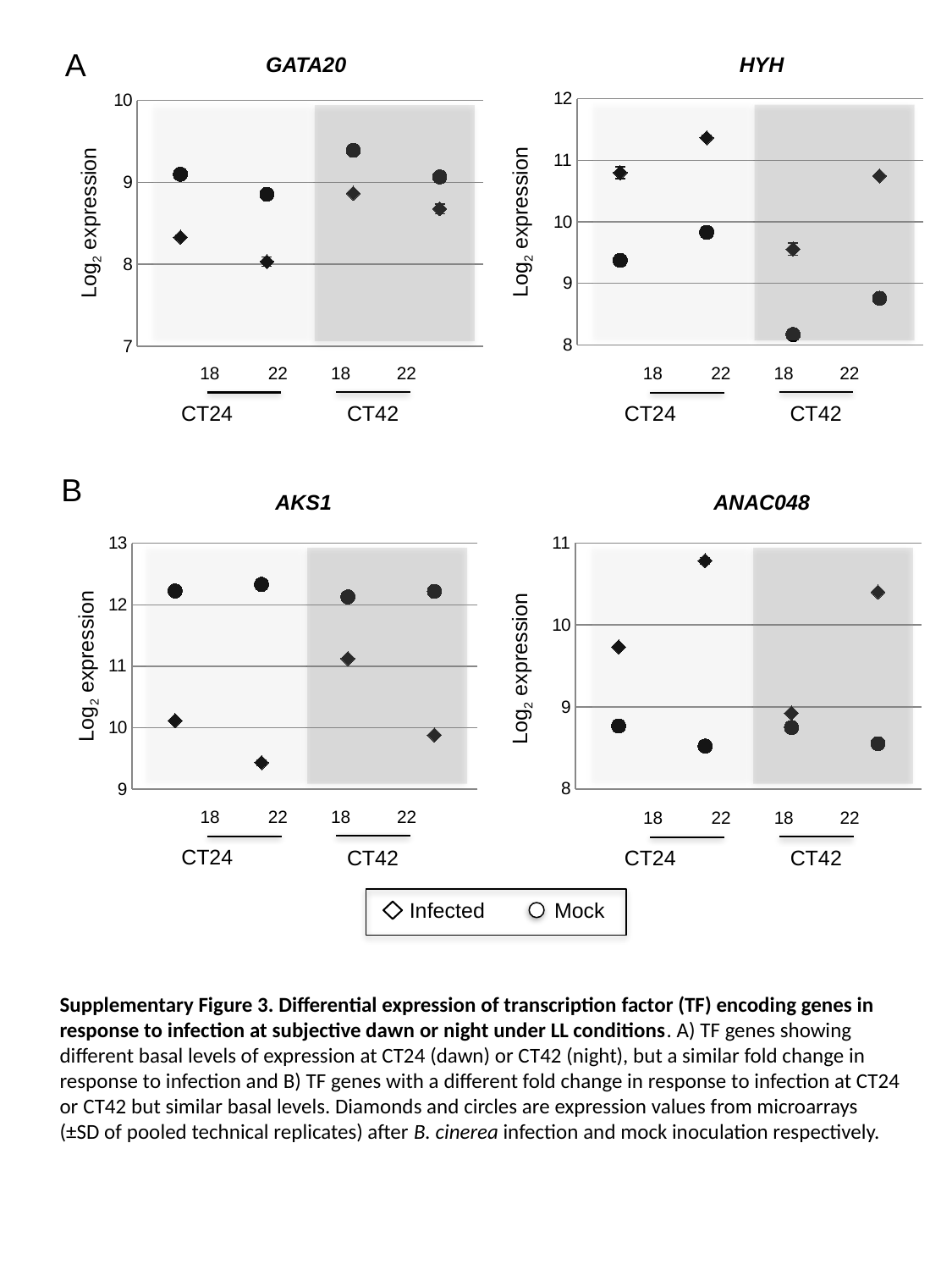

A
GATA20
HYH
### Chart
| Category | infected | mock |
|---|---|---|
### Chart
| Category | Infected | Mock |
|---|---|---|
Log2 expression
Log2 expression
18
22
18
22
18
22
18
22
CT24
CT24
CT42
CT42
B
AKS1
ANAC048
### Chart
| Category | infected | mock |
|---|---|---|
### Chart
| Category | infected | mock |
|---|---|---|
Log2 expression
Log2 expression
18
22
18
22
18
22
18
22
CT24
CT42
CT24
CT42
Mock
Infected
Supplementary Figure 3. Differential expression of transcription factor (TF) encoding genes in response to infection at subjective dawn or night under LL conditions. A) TF genes showing different basal levels of expression at CT24 (dawn) or CT42 (night), but a similar fold change in response to infection and B) TF genes with a different fold change in response to infection at CT24 or CT42 but similar basal levels. Diamonds and circles are expression values from microarrays (±SD of pooled technical replicates) after B. cinerea infection and mock inoculation respectively.
