# Supplementary material for: Jasmonate signalling drives time‐of‐day differences in susceptibility of Arabidopsis to the fungal pathogen Botrytis cinerea
Source: Plant J. 2015 Nov 21;84(5):937–48. doi: 10.1111/tpj.13050 (PMC4982060; doi:10.1111/tpj.13050)
Supplement: Supplementary file 4 — Figure S4. JAZ6 expression is transiently induced during B. cinerea infection. [file TPJ-84-937-s004.pptx]

## Slide 1
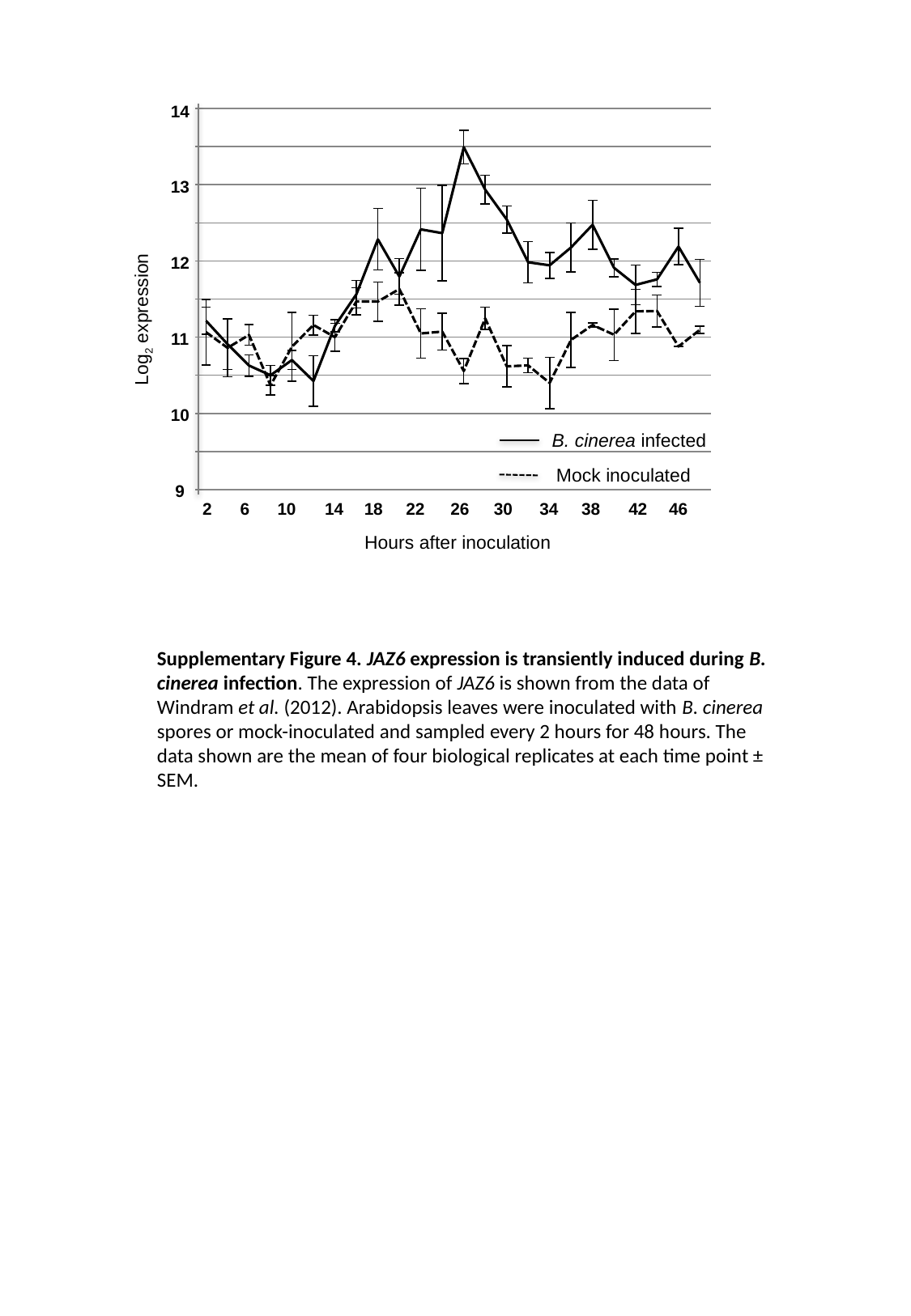

14
### Chart
| Category | | |
|---|---|---|13
12
Log2 expression
11
10
9
2
6
10
 14
18
22
26
30
34
38
42
46
Hours after inoculation
B. cinerea infected
Mock inoculated
Supplementary Figure 4. JAZ6 expression is transiently induced during B. cinerea infection. The expression of JAZ6 is shown from the data of Windram et al. (2012). Arabidopsis leaves were inoculated with B. cinerea spores or mock-inoculated and sampled every 2 hours for 48 hours. The data shown are the mean of four biological replicates at each time point ± SEM.
