# Supplementary material for: Jasmonate signalling drives time‐of‐day differences in susceptibility of Arabidopsis to the fungal pathogen Botrytis cinerea
Source: Plant J. 2015 Nov 21;84(5):937–48. doi: 10.1111/tpj.13050 (PMC4982060; doi:10.1111/tpj.13050)
Supplement: Supplementary file 5 — Figure S5. Stomata are not a primary point of entry for B. cinerea hyphae during infection of Arabidopsis. [file TPJ-84-937-s005.pptx]

## Slide 1
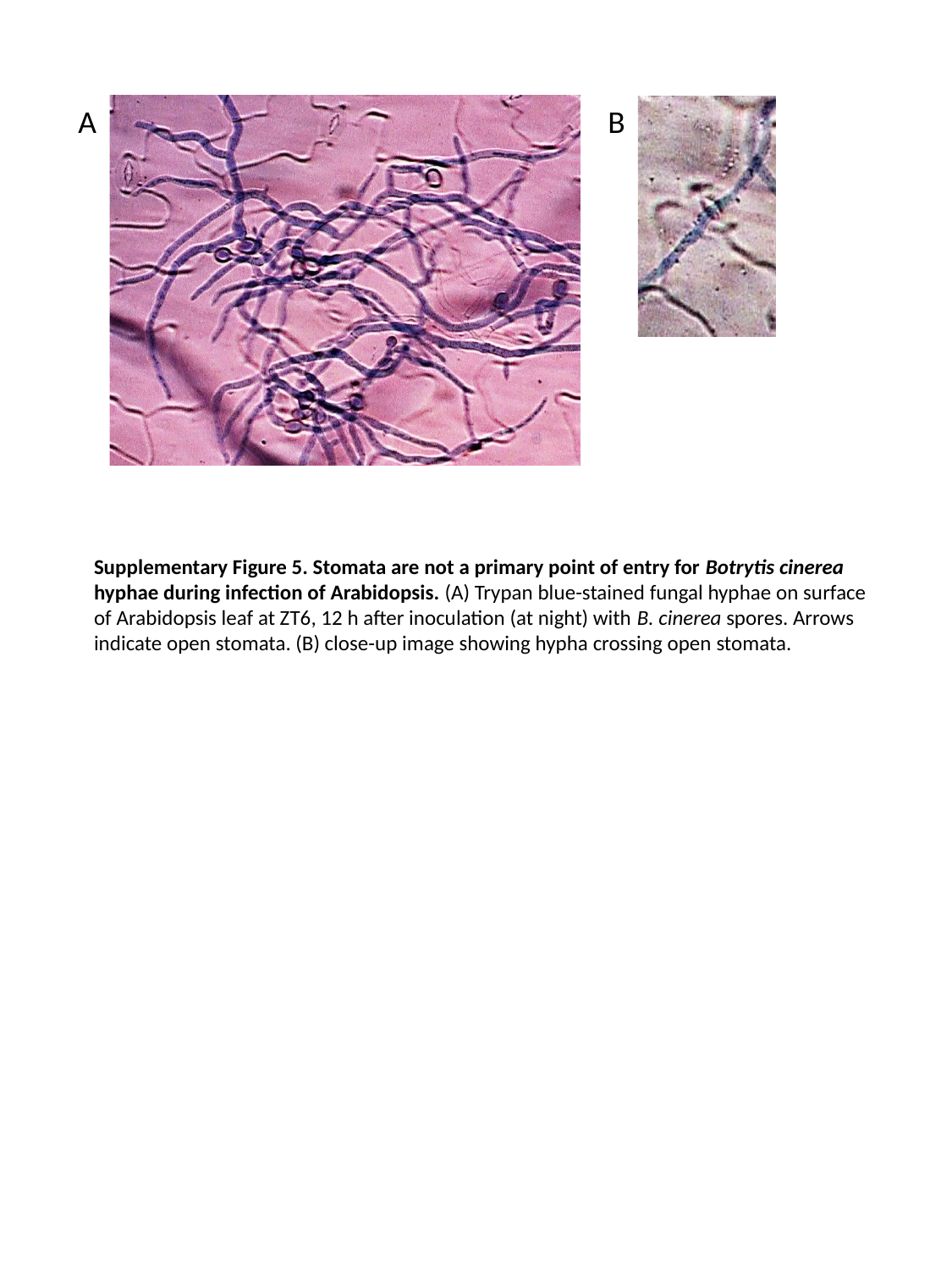

A
B
Supplementary Figure 5. Stomata are not a primary point of entry for Botrytis cinerea hyphae during infection of Arabidopsis. (A) Trypan blue-stained fungal hyphae on surface of Arabidopsis leaf at ZT6, 12 h after inoculation (at night) with B. cinerea spores. Arrows indicate open stomata. (B) close-up image showing hypha crossing open stomata.
