# Supplementary material for: Jasmonate signalling drives time‐of‐day differences in susceptibility of Arabidopsis to the fungal pathogen Botrytis cinerea
Source: Plant J. 2015 Nov 21;84(5):937–48. doi: 10.1111/tpj.13050 (PMC4982060; doi:10.1111/tpj.13050)
Supplement: Supplementary file 6 — Figure S6. Expression of JAZ6 in the jaz6 mutant line and Col‐0 18 hpi with B. cinerea or mock control. [file TPJ-84-937-s006.pptx]

## Slide 1
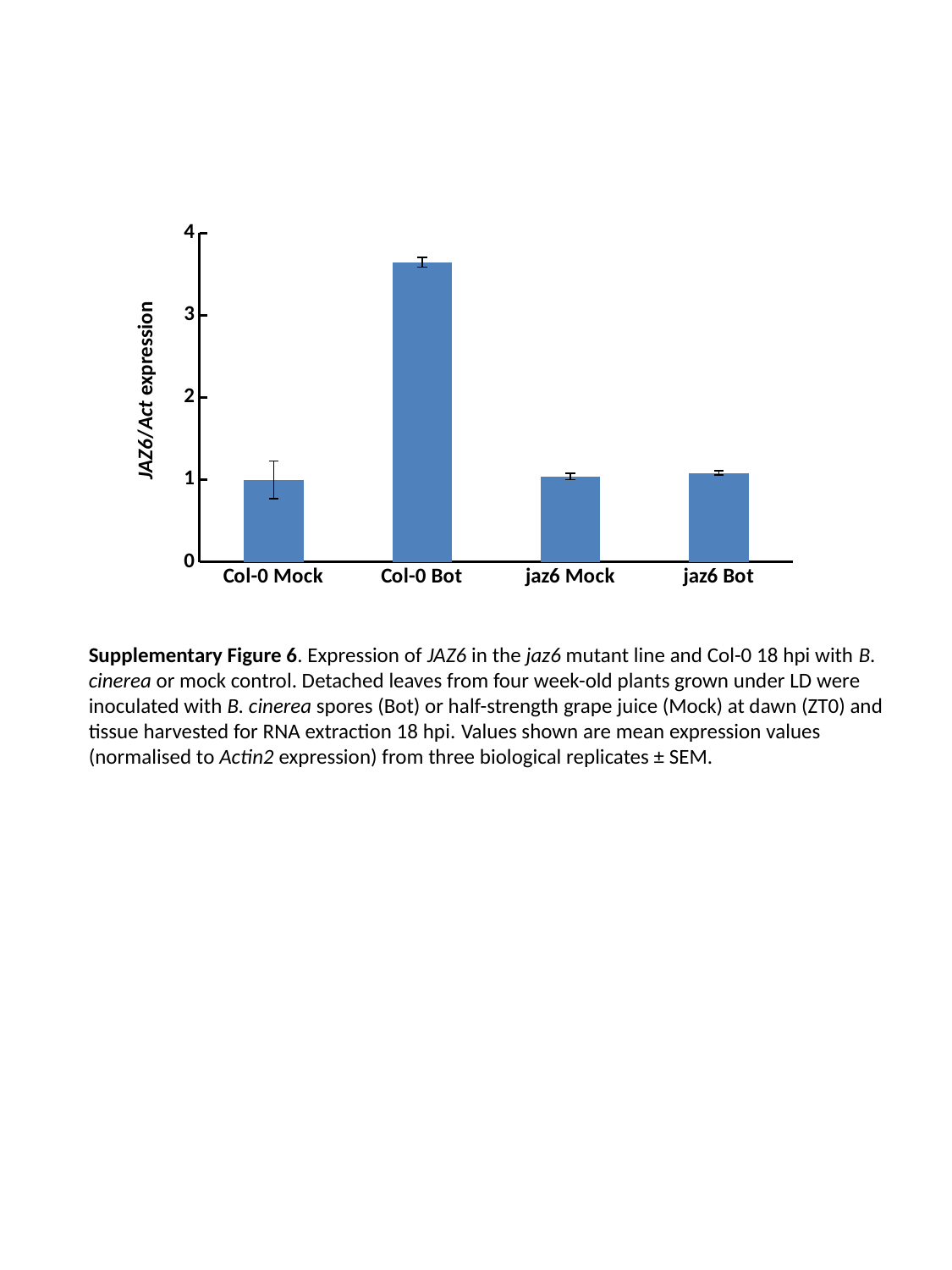

### Chart
| Category | |
|---|---|
| Col-0 Mock | 0.99741984005101 |
| Col-0 Bot | 3.64591399476231 |
| jaz6 Mock | 1.040916123475508 |
| jaz6 Bot | 1.08400306501347 |JAZ6/Act expression
Supplementary Figure 6. Expression of JAZ6 in the jaz6 mutant line and Col-0 18 hpi with B. cinerea or mock control. Detached leaves from four week-old plants grown under LD were inoculated with B. cinerea spores (Bot) or half-strength grape juice (Mock) at dawn (ZT0) and tissue harvested for RNA extraction 18 hpi. Values shown are mean expression values (normalised to Actin2 expression) from three biological replicates ± SEM.
