# Supplementary material for: Jasmonate signalling drives time‐of‐day differences in susceptibility of Arabidopsis to the fungal pathogen Botrytis cinerea
Source: Plant J. 2015 Nov 21;84(5):937–48. doi: 10.1111/tpj.13050 (PMC4982060; doi:10.1111/tpj.13050)
Supplement: Supplementary file 7 — Table S1. Primers used in quantitative PCR experiments. [file TPJ-84-937-s007.docx]

| Gene | Primer sequence | Annealing temp (°C) | Amplicon size (bp) |
| --- | --- | --- | --- |
| *Actin-2* (At3g18780) | AGTGGTCGTACAACCGGTATTGT (900 nM)  CATGAGGTAATCAGTAAGGTCACGT (300 nM) | 60 | 138 |
| *BT2* (At3g48360) | TTGCAAGCGGATGCTTCAAC  CCCGTTTCCTAAATTGCCTGC | 60 | 98 |
| *ERF6* (At4g17490) | CGGTTGTAGCAGCAGAGGAG  AACACGAGTTCCACGACGAG | 58 | 107 |
| *JAZ6* (At1g72450) | ACAGGGCTGTGGCTAGAG  CTTTCTTGTCCACCTCCATC | 55 | 171 |
| *PDF1.1* (At1g75830) | CTGCTCTTGAAGCACCGATGG  CATGTCGTGCTTTCTCAAGGTT | 60 | 132 |
| *PDF1.3* (At2g26010) | AATATAATCATGGCTAAGTCTGCTG  TCCACCATTATCGGTGCTTCA | 58 | 92 |
| *PUX1* (At3g27310) | TTTTTACCGCCTTTTGGCTA  ATGTTGCCTCCAATGTGTGA | 60 | 149 |
| *RAP2.7* (At2g28550) | GCGTGGAGTTAGCTTGAGGATA  TCTTCCCTCGGCGGGATTAT | 60 | 136 |
| *SPL2* (At5g43270) | CCTCACTGTCAAGTTGAAGGC  CTACGCCACTCACAACGACT | 60 | 118 |
| *B. cinerea* beta tubulin (XM_001560987) | TTCCATGAAGGAGGTTGAGG  TACCAACGAAGGTGGAGGAC | 60 | 146 |

**Supplementary Table 1.** Primers used in quantitative PCR experiments. Final concentration (if different from 200 nm), annealing temperature used in qPCR and resulting amplicon size are shown.
