# Supplementary material for: Jasmonate signalling drives time‐of‐day differences in susceptibility of Arabidopsis to the fungal pathogen Botrytis cinerea
Source: Plant J. 2015 Nov 21;84(5):937–48. doi: 10.1111/tpj.13050 (PMC4982060; doi:10.1111/tpj.13050)
Supplement: Supplementary file 8 — Methods S1. Grouping of the differentially expressed genes for Gene Ontology and motif analysis. [file TPJ-84-937-s008.docx]

Supporting Information Method S1

**Grouping of the differentially expressed genes for Gene Ontology and motif analysis:**

The 901 genes were grouped according to whether they were upregulated (≥log_2_0.6) or downregulated (<=log_2_-0.6) in response to inoculation with *B. cinerea* at subjective dawn. If there was no significant change in expression after inoculation at subjective dawn, then the change in expression after inoculation at subjective night was used. Only 12 genes showed both up- and downregulation after inoculation at subjective dawn (i.e. direction of expression change varied between 18 and 22 hpi); these genes were excluded from further analysis. The up- and downregulated genes were split according to the difference in level of expression after inoculation at subjective dawn and night (at the same time point), or if this did not change ≥log_2_0.6, then the difference in the ratio of infected:mock at subjective dawn compared to night. This resulted in eight groups of genes: upregulated in response to *B. cinerea* and upregulated after inoculation at subjective dawn compared to inoculation at night by 18 hpi (UPUP18); upregulated in response to *B. cinerea* and upregulated after inoculation at subjective dawn compared to inoculation at subjective night only at 22 hpi (UPUP22); downregulated in response to *B. cinerea* and downregulated after inoculation at subjective dawn compared to inoculation at subjective night by 18 hpi (DOWNDOWN18); downregulated in response to *B. cinerea* and downregulated after inoculation at subjective dawn compared to inoculation at subjective night only at 22 hpi (DOWNDOWN22); upregulated in response to *B. cinerea* but downregulated after inoculation at subjective dawn compared to inoculation at subjective night by 18 hpi (UPDOWN18); upregulated in response to *B. cinerea* and downregulated after inoculation at subjective dawn compared to inoculation at subjective night only at 22 hpi (UPDOWN22); downregulated in response to *B. cinerea* but upregulated after inoculation at subjective dawn compared to inoculation at subjective night by 18 hpi (DOWNUP18); downregulated in response to *B. cinerea* and upregulated after inoculation at subjective dawn compared to inoculation at night only at 22 hpi (DOWNUP22).
