# Supplementary material for: Jasmonate signalling drives time‐of‐day differences in susceptibility of Arabidopsis to the fungal pathogen Botrytis cinerea
Source: Plant J. 2015 Nov 21;84(5):937–48. doi: 10.1111/tpj.13050 (PMC4982060; doi:10.1111/tpj.13050)
Supplement: Supplementary file 14 [file TPJ-84-937-s014.docx]

**SUPPORTING INFORMATION**

**Supplementary Figure 1. Growth of *Botrytis cinerea* is restricted in plants inoculated at subjective dawn versus subjective night. Detached leaves were inoculated with *B. cinerea* spores at CT24 (subjective dawn) or CT42 (subjective night) under LL conditions, and harvested at 72 hpi. *B. cinerea* tubulin expression was determined using qPCR, with normalization to host *PUX1* expression levels. Data shown are mean values ± SEM from three biological repeats.**

**Supplementary Figure 2: Diagram illustrating selection of differentially expressed genes. Genes that showed a different fold change in expression in response to inoculation at subjective dawn and night but no subsequent difference in expression level (e.g. A), genes with a similar fold change in response to infection at dawn or night but different levels of expression after infection (e.g. B) and genes with different fold change and level of expression in response to infection at subjective dawn and night (e.g. C and D) were selected.**

**Supplementary Figure 3. Differential expression of transcription factor (TF) encoding genes in response to infection at subjective dawn or night under LL conditions. A) TF genes showing different basal levels of expression at CT24 (dawn) or CT42 (night), but a similar fold change in response to infection and B) TF genes with a different fold change in response to infection at CT24 or CT42 but similar basal levels. Diamonds and circles are expression values from microarrays (±SD of pooled technical replicates) after *B. cinerea* infection and mock inoculation respectively.**

**Supplementary Figure 4. *JAZ6* expression is transiently induced during *B. cinerea* infection. The expression of *JAZ6* is shown from the data of Windram *et al.* (2012). Arabidopsis leaves were inoculated with *B. cinerea* spores or mock-inoculated and sampled every 2 hours for 48 hours. The data shown are the mean of four biological replicates at each time point ± SEM.**

**Supplementary Figure 5. Stomata are not a primary point of entry for *Botrytis cinerea* hyphae during infection of Arabidopsis. (A) Trypan blue-stained fungal hyphae on surface of Arabidopsis leaf at ZT6, 12 h after inoculation (at night) with *B. cinerea* spores. Arrows indicate open stomata. (B) Close-up image showing hypha crossing open stomata.**

**Supplementary Figure 6. Expression of *JAZ6* in the *jaz6* mutant line and Col-0 18 hpi with *B. cinerea* or mock control. Detached leaves from four week-old plants grown under LD were inoculated with *B. cinerea* spores (Bot) or half-strength grape juice (Mock) at dawn (ZT0) and tissue harvested for RNA extraction 18 hpi. Values shown are mean expression values (normalised to *Actin2* expression) from three biological replicates ± SEM.**

**Supplementary Data set 1**. Expression data 18 and 22 hpi after subjective dawn and night inoculations with *B. cinerea* for genes with consistent expression patterns in previous studies.

**Supplementary Data set 2:** Arabidopsis genes whose expression changes significantly in response to *B. cinerea* and to the time of day at which inoculation occurred.

**Supplementary Data set 3**. Transcription factor genes that are differentially expressed in response to inoculation with *B. cinerea* at different times of the day.

**Supplementary Data set 4.** Biological Process Gene Ontology terms significantly overrepresented in groups of genes differentially expressed in response to inoculation at different times of the day.

**Supplementary Data set 5.** Motifs significantly overrepresented in upstream regions of groups of genes differentially expressed in response to inoculation at different times of the day.

**Supplementary Table 1.** Primers used in quantitative PCR experiments.

**Supplementary Method S1.** Grouping of the differentially expressed genes for Gene Ontology and motif analysis.
